# Total and differential white blood cell count in cannabis users: results from the cross-sectional National Health and Nutrition Examination Survey, 2005–2016

**DOI:** 10.1186/s42238-019-0007-8

**Published:** 2019-07-09

**Authors:** Omayma Alshaarawy

**Affiliations:** Department of Family Medicine, Michigan State University, 788 Service Road, East Lansing, MI 48824 USA

**Keywords:** Cannabis, NHANES, White blood cells

## Abstract

**Background:**

Elevated white blood cell (WBC) count in tobacco cigarette smokers compared to non-smokers has been well documented, but little is known on circulating WBC counts and cannabis use.

**Methods:**

The National Health and Nutrition Examination Survey (2005–2016) is designed to be nationally representative of United States non-institutionalized population**.** The current study includes adult participants 20–59 years of age (*n* = 16,430) who underwent a detailed examination in the mobile examination center (MEC). Cannabis use was measured using Audio Computer-Assisted Self-Interview. Cannabis use was classified into never, former, occasional (1–7 days of the past 30 days), and heavy (> 7 days of the past 30 days). WBC count was measured using the Coulter Counter method.

**Results:**

Total WBC count was higher among heavy cannabis users when compared to never users (β = 189; 95% confidence interval: 74, 304, *p* = 0.001). Among circulating WBC types, modest differences were observed for neutrophil count. Neither former nor occasional cannabis use was associated with total or differential WBC counts.

**Conclusions:**

A modest association between heavy cannabis use and WBC count was detected. Additional research is needed to understand the immune related effects of different modes of cannabis use and to elucidate the role of proinflammatory chemicals generated from smoking cannabis.

**Electronic supplementary material:**

The online version of this article (10.1186/s42238-019-0007-8) contains supplementary material, which is available to authorized users.

## Background

White blood cells (WBCs) are a heterogeneous group of nucleated cells that function mainly as immune cells. WBCs originate in the bone marrow, and can be classified into granulocytes (neutrophils, eosinophils and basophils), and agranulocytes (lymphocytes and monocytes). Cigarette smoking generates several chemicals that are implicated in oxidative stress pathways and systemic inflammation (Lee et al., 2012). Elevated WBC count in tobacco cigarette smokers have been well documented (Higuchi et al., 2016; Jensen et al., 1998), whereas tobacco abstinence is associated with sustained decrease in WBC count (Abel et al., 2005). While the prevalence of tobacco smoking is decreasing (Wang et al., 2018), the use of other combustible products such as cannabis is increasing in the United States (Grucza et al., 2016).

Cannabis mediate its effects through a number of G-protein-coupled receptors, importantly cannabinoid-1 (CB1) and cannabinoid-2 (CB2) receptors. Cannabinoid-2 receptors are expressed in various components of the immune system including bone marrow, thymus, tonsils and spleen, whereas CB1 receptors are highly expressed in the central nervous system, and at lower levels in the immune system (Pertwee et al., 2010).

Human B-lymphocytes, monocytes, neutrophils, and T-lymphocytes express cannabinoid receptors with varying degrees (Galiègue et al., 1995). Laboratory studies have demonstrated the effects of cannabinoids on hematopoiesis, and immune cell proliferation using animal and cell based models (Nagarkatti et al., 2009). Additionally, several studies have examined the association of cannabis use and WBCs in human immunodeficiency virus (HIV) infected and uninfected research participants. Lorenz et al. have reported elevated WBC count in HIV positive men who used cannabis (Lorenz et al., 2017). In a randomized controlled trial, neither CD4 nor CD8 cell count was affected by cannabis use (Abrams et al., 2003). Similarly, in prospective studies cannabis use was not associated with CD4 and CD8 cell counts (Chao et al., 2008; Marcellin et al., 2017). Conversely, other studies reported an increase in circulating CD4 cells in cannabis users (Thames et al., 2016; Montarroyos et al., 2014; Keen et al., 2019).

Research on cannabis use and the immune system in the general population is scarce (Friedman et al., 1990; Rajavashisth et al., 2012). The 2018 report of the National Academies of Sciences, Engineering and Medicine identified the lack of studies on cannabis use and immunity in healthy individuals as a research gap (National Academies of Sciences, 2017).

The aim of the current study is to estimate the association of cannabis use and WBC count, an important component of the immune system and a predictor of mortality, cardiovascular disease, and cancer (Margolis et al., 2005; Twig et al., 2013; Willems et al., 2010). To test the hypothesis that cannabis use is associated with elevated WBC count, data from the United States (US) National Health and Nutrition Examination Survey are analyzed.

## Methods

### Study population

The National Health and Nutrition Examination Survey (NHANES) is a series of ongoing cross-sectional surveys, designed to yield nationally representative estimates for the US non-institutionalized civilian population through multistage area probability sampling (United States Centers for Disease Control and Prevention, 2010). The current study estimates are derived by combining NHANES survey cycles 2005–6, 2007–8, 2009–10, 2011–12, 2013–14 and 2015–16. The study protocol has been reviewed and approved by the National Center for Health Statistics institutional review board and informed consents were obtained from all participants. The data analyzed in the current study are publically available in the National Center for Health Statistics data repository, https://wwwn.cdc.gov/nchs/nhanes/Default.aspx.

The current study was limited to men and non-pregnant women aged 20 to 59 years who attended the physical examination in NHANES Mobile Examination Center (*n* = 18,687). Participants with missing data on WBC count (*n* = 724), with WBC count outside the normal range (4000–11,000 cells/uL) suggesting acute infection or other pathological processes (*n* = 1431), or with missing data on covariates (*n* = 93) were not included in the current analyses. Those who were excluded were more likely to be non-Hispanic Blacks, daily tobacco cigarette smokers with high school education or less. Approximately 37% of those excluded never used cannabis, 45% were former users, 9% were occasional users and 9% were heavy users (Data are not shown in table/figure).

### Outcome

The Laboratory methods used have been previously described in the NHANES Laboratory Procedures Manual (United States Center for Disease Control and Prevention, 2006). Briefly, blood (3–4 mL) was collected in K3 EDTA tube following established venipuncture protocol and procedures. A complete blood count with 5-part differential was performed in duplicate. The methods used to derive WBC count are based on the Coulter method of counting and sizing. The differential WBC count is based on Volume, Conductivity and Scatter technology.

### Exposure

The variable of central interest is cannabis use, assessed via self-report responses to standardized questions using an Audio Computer-Assisted Self-Interview (ACASI) system at NHANES mobile examination center (MEC). The ACASI approach is intended to promote accuracy and completeness of reporting on sensitive topics (Estes et al., 2010).

Participants were classified as never users, former users (used cannabis at least once in lifetime but not in the 30 days prior to the exam), and recent users (used cannabis at least once in the 30 days prior to the exam). Recent users were further classified by the number of days of use in the 30 days prior to the interview into occasional (≤50th percentile, used cannabis 1–7 days) and heavy users (>50th percentile, used cannabis 8–30 days).

### Other variables

Guided by previous studies (Anthony et al., 2016)**,** sociodemographic characteristics (age, sex, race/ethnicity, and education), habits associated with cannabis use and WBC count (alcohol drinking and tobacco cigarette smoking), and body mass index (BMI) were classified as potential confounders. The Alcohol use questionnaire was administered in NHANES MEC using Computer-Assisted Personal Interviewing (CAPI) system focusing on lifetime and past 12-month alcohol use. Alcohol drinking was categorized into non-drinking, occasional drinking (< 1 drink/day in the 12 months prior to the exam), or daily drinking (≥1 drink/day in the 12 months prior to the exam). Cigarette smoking questions were asked in the participant household using CAPI. Participants were asked if they ever smoked 100 cigarettes in lifetime and those who answered yes were asked if they now smoke everyday, on some days, or not at all. An answer of ‘everyday’ qualified the participant as a daily tobacco cigarette smoker, and an answer of “somedays” qualified the participant as an occasional smoker. Participants who smoked < 100 cigarettes in their lifetime were classified as never smokers whereas participants who smoked 100+ cigarettes in lifetime and did not smoke at the time of the interview were classified as former smokers. BMI was measured in the MEC as weight in kilograms divided by height in meters squared.

### Statistical analysis

Descriptive statistics were used to compare selected characteristics of the study participants by cannabis use status. Linear regression modelling was then used to estimate differences in cell counts by cannabis use status. First unadjusted estimates were obtained. Models were then adjusted for age and sex, followed by adjustment for race/ethnicity, education, survey cycle, body mass index, alcohol drinking, and tobacco cigarette smoking. Sensitivity analyses were then used to investigate if significant associations were still present 1) using different cutoffs for heavy cannabis use, 2) excluding participants with a history of chronic medical conditions, 3) including participants with low (< 4000 cells/uL) or high (> 11,000 cells/uL) WBC count, and 4) adjusting for serum cotinine levels. NHANES examination weights that account for the unequal probabilities of selection, oversampling and non-response were applied to all analyses and standard errors were estimated using the Taylor series linearization method. Statistical significance was established at *p* = 0.05 for total WBC count analyses whereas a *p*-value of 0.01 was used for differential WBC analyses to adjust for multiple testing (0.05/5 tests). Analyses were conducted using SAS (V.9.4) software.

## Results

The majority of participants reported ever use of cannabis, with 13% reporting cannabis use in the 30 days prior to the interview (Table 1). Compared to never users, recent users were more likely to be younger, males, and daily tobacco cigarette smokers.

**Table 1:** Selected Charcteristics of the Study Participants by Cannabis Use Status. Data for the US NHANES, 2005–2016

Cannabis use status	Never (*n* = 7649)	Former (*n* = 6608)	Occasional (*n* = 1109)	Heavy (*n* = 1064)	*p*-value^a^
Selected charcteristics	Mean (SE) or unweighted n (weighted column%)^b^				
Age (years)	40 (0.2)	41 (0.2)	35 (0.6)	35 (0.6)	< 0.0001
Female	4302 (55%)	3055 (47%)	450 (39%)	327 (32%)	< 0.0001
Race/ethnicity					
White	2255 (55%)	3510 (76%)	501 (66%)	530 (70%)	< 0.0001
Black	1346 (11%)	1376 (10%)	329 (17%)	298 (15%)	
Hispanic	2940 (24%)	1270 (10%)	194 (12%)	159 (10%)	
All others	1108 (11%)	452 (4%)	85 (6%)	77 (5%)	
Education					
< high school	1984 (18%)	1027 (11%)	234 (16%)	256 (20%)	< 0.0001
High school	1540 (20%)	1491 (21%)	291 (25%)	305 (27%)	
> high school	4125 (62%)	4090 (68%)	584 (59%)	503 (53%)	
Tobacco cigarette smoking					
Never	6012 (81%)	2920 (45%)	376 (33%)	228 (21%)	< 0.0001
Former	823 (10%)	1747 (29%)	174 (20%)	189 (21%)	
Occasional	213 (2%)	380 (5%)	108 (10%)	104 (9%)	
Daily	601 (7%)	1561 (21%)	451 (37%)	543 (48%)	
Alcohol drinking					
Non-drinker	2872 (34%)	1124 (15%)	76 (6%)	97 (8%)	< 0.0001
Occasional	4460 (64%)	5231 (81%)	970 (88%)	902 (85%)	
Daily	117 (2%)	253 (4%)	63 (6%)	65 (7%)	
Survey cycle					
2005–06	1010 (16%)	1035 (17%)	143 (15%)	124 (15%)	0.05
2007–08	1239 (16%)	1234 (18%)	173 (15%)	153 (13%)	
2009–10	1453 (17%)	1176 (16%)	202 (16%)	200 (16%)	
2011–12	1236 (16%)	1069 (17%)	177 (17%)	181 (17%)	
2013–14	1337 (17%)	1155 (17%)	216 (17%)	208 (19%)	
2015–16	1374 (18%)	939 (15%)	198 (20%)	198 (21%)	
BMI (Kg/m^2^)	29 (0.1)	29 (0.1)	27 (0.2)	27 (0.2)	< 0.0001

^a^ P-value for global test: ANOVA for continuous variables and Pearson χ2 tests for categorical variables

^b^ Percentages may not add up to 100% due to rounding

The crude and age-sex-adjusted mean WBC count were higher among cannabis users compared to never users (Table 2). After adjusting for potential confounding variables, only heavy users had higher WBC count (β = 189; 95% confidence interval: 74, 304, *p* = 0.001) when compared to never users. Among the differential subpopulations, modest differences were observed for neutrophil counts (β = 172; 95% CI = 44, 299, *p* = 0.001). In addition, heavy cannabis users had higher monocyte count, yet the association failed to reach statistical significance at the adjusted *p* < 0.01. Neither former nor occasional cannabis use was associated with total or differential WBC counts.

**Table 2:** Association of Cannabis use, and Total and Differential WBC Count. Data for the US NHANES, 2005–2016

Cannabis use status	Never	Former	*p*- value^c^	Occasional	*p*- value^c^	Heavy	*p*- value^c^
Total WBC count (cells/uL)							
Crude β (95% CI)	0 (referent)	**83 (16, 150)**	**0.02**	**140 (−6, 286)**	**0.06**	**463 (343, 583)**	**< 0.0001**
Age-sex adjusted β (95% CI)^a^	0 (referent)	**99 (31, 167)**	**0.005**	**149 (3, 294)**	**0.04**	**477 (359, 595)**	**< 0.0001**
Multivariable adjusted β (95% CI)^b^	0 (referent)	−26 (−94, 43)	0.46	−36 (− 170, 98)	0.59	189 (74, 304)	**0.001**
Lymphocyte count (cells/uL)							
Crude β (95% CI)	0 (referent)	−3 (− 37, 30)	0.79	49 (− 31, 130)	0.11	**111 (44, 179)**	**< 0.0001**
Age-sex adjusted β (95% CI)^a^	0 (referent)	7 (−26, 39)	0.58	36 (−43, 115)	0.23	**99 (35, 163)**	**< 0.0001**
Multivariable adjusted β (95% CI)^b^	0 (referent)	−14 (−45, 18)	0.25	−29 (− 106, 48)	0.33	11 (−57, 79)	0.68
Monocyte count (cells/uL)							
Crude β (95% CI)	0 (referent)	**15 (7, 24)**	**< 0.0001**	**33 (14, 53)**	**< 0.0001**	**55 (36, 73)**	**< 0.0001**
Age-sex adjusted β (95% CI)^a^	0 (referent)	**11 (2, 19)**	**0.001**	**23 (3, 43)**	**0.003**	**40 (22, 58)**	**< 0.0001**
Multivariable adjusted β (95% CI)^b^	0 (referent)	−1 (−10, 7)	0.66	7 (− 11, 26)	0.30	18 (− 1, 37)	0.01
Neutrophil count (cells/uL)							
Crude β (95% CI)	0 (referent)	62 (−13, 137)	0.03	40 (− 103, 184)	0.46	**289 (164, 414)**	**< 0.0001**
Age-sex adjusted β (95% CI)^a^	0 (referent)	75 (−2, 151)	0.01	76 (−66, 219)	0.16	**335 (210, 460)**	**< 0.0001**
Multivariable adjusted β (95% CI)^b^	0 (referent)	−12 (−94, 70)	0.70	− 18 (− 156, 120)	0.74	**172 (44, 299)**	**0.001**
Basophil count (cells/uL)							
Crude β (95% CI)	0 (referent)	2 (−1, 5)	0.09	4 (−2, 9)	0.09	6 (− 1, 12)	0.02
Age-sex adjusted β (95% CI)^a^	0 (referent)	2 (− 1, 5)	0.11	5 (− 1, 11)	0.03	**7 (1, 14)**	**0.005**
Multivariable adjusted β (95% CI)^b^	0 (referent)	0 (−3, 3)	0.84	0 (−6, 6)	0.86	1 (− 6, 8)	0.69
Eosinophil count (cells/uL)							
Crude β (95% CI)	0 (referent)	6 (−2, 14)	0.04	**16 (1, 31)**	**0.006**	8 (−8, 25)	0.19
Age-sex adjusted β (95% CI)^a^	0 (referent)	4 (−4, 12)	0.21	12 (−3, 27)	0.04	2 (− 14, 19)	0.69
Multivariable adjusted β (95% CI)^b^	0 (referent)	0 (−9, 8)	0.91	4 (− 12, 21)	0.49	− 8 (− 26, 9)	0.23

^a^ Estimates adjusted for age (years), age squared and sex

^b^ Estimates adjusted for age (years), age squared, sex, race/ethnicity (White, Black, Hispanic, all others), education (<high school, high school, >high school), survey cycle (2005–06, 2007–08, 2009–10, 2011–12, 2013–14, 2015–16), body mass index (Kg/m^2^), alcohol drinking (non-drinker, occasional, daily) and tobacco cigarette smoking (never, former, occasional, daily)

^c^ 95% confidence intervals are presented in the total WBC count analyses whereas 99% confidence intervals are presented in the differential count analyses to adjust for multiple testing

The results did not change when different cutoffs for heavy use were implemented. For example, cannabis use 12–30 days in the 30 days prior to the exam (using the Addiction severity index definition of regular use as 3 times/week) was associated with higher WBC count (β = 217; 95% confidence interval: 94, 340, *p* < 0.001) when compared to never users. Also, our results did not change when participants with history of cardiovascular disease, liver disease, cancer or who tested positive for HIV antibody were excluded (Additional file 1: Table S1), or when participants with low (< 4000 cells/uL) or high (> 11,000 cells/uL) WBC count were included in the analyses.

Additionally, adjusting for serum cotinine levels (available in NHANES 2005–2014 only at the time of analyses) as a measure of secondhand smoke exposure or as a measure of nicotine exposure if cannabis is mixed with tobacco did not change our conclusions (Fairman and Alshaarawy, 2017), that is heavy use is associated with total WBC and neutrophil counts (Additional file 1: Table S2).

Adjustment for tobacco smoking attenuated the estimates appreciably. Exploratory analyses to probe subgroup variation in the estimates disclosed no robust differences by tobacco smoking status. Mean WBC count were higher among heavy cannabis users compared to never users in the tobacco non-smoker subgroup (never and former smokers combined, multivariable adjusted β = 372; 95% CI = 203, 542, *p* < 0.0001) and the tobacco smoker subgroup (occasional and daily smokers combined, multivariable adjusted β = 257; 95% CI = 70, 443, *p* = 0.007).

## Discussion

The main findings of this study may be summarized succinctly as follows. Heavy cannabis use is associated with elevated total WBC count consistent with prior epidemiological studies conducted in the general population (Friedman et al., 1990; Rajavashisth et al., 2012). These studies, however, did not investigate the association of cannabis use and WBC differential counts. In the current study, neutrophils the most abundant cells of the innate immune response, had the strongest association followed by monocytes but not lymphocytes, eosinophils and basophils. These associations were evident when participants with HIV or other chronic conditions were excluded from the analyses.

Before detailed discussion of these results, several of the important study limitations merit attention. The observational nature of the study constrained causal inferences. Even though NHANES collects blood and urine specimens, drug testing is not conducted, and cannabis use was self-reported which may lead to non-differential misclassification bias. There was no available information on the route of administration of cannabis (smoking, ingestion, etc.) or cannabis preparation/potency.

Despite limitations such as these, the study findings are of interest because of the large sample size, the NHANES standardized data collection approaches, and the ability to adjust for potential confounders. Cannabis use was quantified according to the number of use days in an interval that does not require long recall (past 30 days). In addition, the study is based on fairly recent NHANES surveys (2005–16) which might be more representative of the increasing cannabis potency compared to NHANES III (1988–1994) surveys (Rajavashisth et al., 2012; ElSohly et al., 2016).

Laboratory studies reported suppression of immune responses with cannabinoid administration (Klein, 2005), and some epidemiological studies found lower levels of inflammatory biomarkers such as fibrinogen, C-reactive protein and interleukin-6 in adult cannabis users (Alshaarawy and Anthony, 2015; Keen et al., 2014; Rajavashisth et al., 2012; Alshaarawy et al., 2019). Conversely, studies in adolescence and young adults indicated that cannabis use is not associated lowered immune responses (Ferguson et al., 2019; Costello et al., 2013). The reported anti-inflammatory effects of cannabis were greatly attenuated when body weight is controlled for. This suggests that the inverse cannabis-body weight association might explain the lower levels of circulating inflammatory biomarkers in adult cannabis users (Penner et al., 2013; Le Strat and Le Foll, 2011; Alshaarawy and Anthony, 2019), given the strong association of inflammation and adiposity (Esser et al., 2014). The results of the current study indicate elevated WBC count among heavy cannabis users, and persisted after adjusting for BMI. This increase might be related to the inflammatory effects of combustion by-products as the most common mode of cannabis use is smoking (Grotenhermen, 2003). The association of cannabis and WBCs was evident in heavy users only, which might indicate increased exposure to proinflammatory chemicals generated from smoking (Wei et al., 2016).

These alterations of immune responses by cannabis use might be associated with increased susceptibility to infections and hence the higher WBC count. Indeed, Tsai et al. have reported an association between regular cannabis use and suboptimal self-rated health status, independent of tobacco smoking (Tsai et al., 2017). Yet, it is possible that the elevated WBC and suboptimal health status contributed to cannabis use rather than cannabis use caused suboptimal health. This hypothesis, though, cannot be tested as NHANES does not collect information on cannabis use motives. Another potential mechanism can be through the effect of cannabinoids on stem cells. Pre-clinical studies suggest that cannabinoids stimulate hematopoiesis (Valk et al., 1997), and hence this stimulation to bone marrow tissues can be associated with increased circulating WBC count in cannabis users.

## Conclusions

Positive associations between heavy cannabis use, and total WBC and neutrophil counts were detected. Clinicians should consider heavy cannabis use in patients presenting with elevated WBC count. Research on heavy cannabis use and cardiovascular health is needed as systemic inflammation, increased cardiovascular risk and increased mortality risk have been all associated with WBC elevation within the normal physiologic range (Lee et al., 2001). Additionally, studies with repeated measures are needed to study immunomodulatory changes in cannabis users, and whether the mode of cannabis use can differentially affect immune responses.

## Additional file

Additional file 1: **Table S1**. Association of Cannabis use, and Total and Differential WBC Count Excluding participants with HIV or chronic conditions^a^. Data for the US NHANES, 2005–2016. **Table S2**. Association of Cannabis use, and Total and Differential WBC Count Additionally Adjusting for Serum Cotinine. Data for NHANES 2005–2014. (DOCX 18 kb)

## Data Availability

The datasets analyzed during the current study are publically available in the National Center for Health Statistics data repository, https://wwwn.cdc.gov/nchs/nhanes/Default.aspx

## Abbreviations

BMI: Body mass index
CB1: Cannabinoid-1
CB2: Cannabinoid-2
CI: Confidence interval
NHANES: National Health and Nutrition Examination Survey
WBC: White blood cell
