# Supplementary material for: Total and differential white blood cell count in cannabis users: results from the cross-sectional National Health and Nutrition Examination Survey, 2005–2016
Source: J Cannabis Res. 2019 Jul 9;1:6. doi: 10.1186/s42238-019-0007-8 (PMC7678768; doi:10.1186/s42238-019-0007-8)
Supplement: Supplementary file 1 — Table S1. Association of Cannabis use, and Total and Differential WBC Count Excluding participants with HIV or chronic conditionsa. Data for the US NHANES, 2005–2016. Table S2. Association of Cannabis use, and Total and Differential WBC Count Additionally Adjusting for Serum Cotinine. Data for NHANES 2005–2014. (DOCX 18 kb) [file 42238_2019_7_MOESM1_ESM.docx]

**Total and differential white blood cell count in cannabis users:**

**Results from the cross-sectional National Health and Nutrition Examination Survey, 2005-2016**

Supplementary tables

| Supplementary Table 1: Association of Cannabis use, and Total and Differential WBC Count Excluding participants with HIV or chronic conditions^a^. Data for the US NHANES, 2005-2016. | | | | | | | |
| --- | --- | --- | --- | --- | --- | --- | --- |
| Cannabis use status | Never, n = 6924 | Former, n = 5641 | *p^c^* | Occasional, n = 937 | *p^c^* | Heavy, n = 916 | *p^c^* |
| Multivariable adjusted β (95% CI)^b^ | | | | | | | |
|  | | | | | | | |
| Total WBC count (cells/uL) | 0 (referent) | -22 (-98, 53) | 0.55 | -1 (-156, 154) | 0.99 | **165 (45, 285)** | **0.03** |
|  | | | | | | | |
| Lymphocyte count (cells/uL) | 0 (referent) | -15 (-50, 19) | 0.24 | -31 (-107, 45) | 0.28 | -1 (-74, 72) | 0.97 |
|  | | | | | | | |
| Monocyte count (cells/uL) | 0 (referent) | -2 (-11, 7) | 0.51 | 8 (-13, 28) | 0.33 | 17 (-2, 37) | 0.02 |
|  | | | | | | | |
| Neutrophil count (cells/uL) | 0 (referent) | -8 (-96, 80) | 0.82 | 20 (-136, 177) | 0.74 | **160 (32, 289)** | **0.001** |
|  | | | | | | | |
| Basophil count (cells/uL) | 0 (referent) | -1 (-4, 3) | 0.68 | -1 (-9, 6) | 0.79 | -1 (-9, 6) | 0.67 |
|  | | | | | | | |
| Eosinophil count (cells/uL) | 0 (referent) | 1 (-7, 9) | 0.74 | 5 (-14, 24) | 0.49 | -8 (-25, 10) | 0.25 |
| a Participants who reported a history of cardiovascular disease, liver conditions, cancer or those who tested positive for HIV antibody were not included in the analyses (n = 2012).  b Estimates adjusted for age (years), age squared, sex, race/ethnicity (White, Black, Hispanic, all others), education (<high school, high school, >high school), survey cycle (2005-06, 2007-08, 2009-10, 2011-12, 2013-14, 2015-16), body mass index (Kg/m^2^), alcohol drinking (non-drinker, occasional, daily) and tobacco cigarette smoking (never, former, occasional, daily).  c 95% confidence intervals are presented in the total WBC count analyses whereas 99% confidence intervals are presented in the differential count analyses to adjust for multiple testing. | | | | | | | |

| Supplementary Table 2: Association of Cannabis use, and Total and Differential WBC Count Additionally Adjusting for Serum Cotinine. Data for NHANES 2005-2014^a^ | | | | | | | |
| --- | --- | --- | --- | --- | --- | --- | --- |
| Cannabis use status | Never, n = 6221 | Former, n = 5631 | *p^c^* | Occasional, n = 903 | *p^c^* | Heavy, n = 857 | *p^c^* |
| Multivariable adjusted β (95% CI)^b^ | | | | | | | |
|  | | | | | | | |
| Total WBC count (cells/uL) | 0 (referent) | -48 (-122, 27) | 0.21 | 12 (-136, 159) | 0.87 | **205 (74, 336)** | **0.002** |
|  | | | | | | | |
| Lymphocyte count (cells/uL) | 0 (referent) | -20 (-54, 14) | 0.12 | -29 (-120, 61) | 0.40 | 13 (-70, 96) | 0.68 |
|  | | | | | | | |
| Monocyte count (cells/uL) | 0 (referent) | -3 (-12, 6) | 0.36 | 3 (-18, 24) | 0.72 | 20 (-1, 41) | 0.02 |
|  | | | | | | | |
| Neutrophil count (cells/uL) | 0 (referent) | -24 (-117, 68) | 0.49 | 31 (-125, 186) | 0.60 | **184 (44, 325)** | **0.001** |
|  | | | | | | | |
| Basophil count (cells/uL) | 0 (referent) | -1 (-4, 3) | 0.69 | 0 (-7, 7) | 0.96 | 1 (-7, 9) | 0.77 |
|  | | | | | | | |
| Eosinophil count (cells/uL) | 0 (referent) | -1 (-10, 7) | 0.66 | 10 (-8, 27) | 0.16 | -8 (-27, 12) | 0.31 |
|  |  |  |  |  |  |  |  |
| a Serum cotinine (ng/mL) is available for NHANES data cycles 2005-2014 only. It was measured by an isotope-dilution high-performance liquid chromatography/atmospheric pressure chemical ionization tandem mass spectrometric method. Geometric mean cotinine levels (ng/mL) were 0.09 (SE = 0.01), 0.62 (SE = 0.1), 6.02 (SE = 1.2), and 19.36 (SE = 3.2) for never, former, occasional and heavy use, respectively.  b Estimates adjusted for age (years), age squared, sex, race/ethnicity (White, Black, Hispanic, all others), education (<high school, high school, >high school), survey cycle (2005-06, 2007-08, 2009-10, 2011-12, 2013-14), body mass index (Kg/m^2^), alcohol drinking (non-drinker, occasional, daily), tobacco cigarette smoking (never, former, occasional, daily) and log-transformed serum cotinine (ng/mL).  c 95% confidence intervals are presented in the total WBC count analyses whereas 99% confidence intervals are presented in the differential count analyses to adjust for multiple testing. | | | | | | | |
